# Association between the caregivers’ oral health literacy and the oral health of children and youth with special health care needs

**DOI:** 10.1371/journal.pone.0263153

**Published:** 2022-01-27

**Authors:** Jagan Kumar Baskaradoss, Aishah AlSumait, Eman Behbehani, Muawia A. Qudeimat

**Affiliations:** 1 Department of Developmental and Preventive Sciences, Faculty of Dentistry, Kuwait University, Safat, Kuwait; 2 School Oral Health Program, Ministry of Health, Kuwait City, Kuwait; Griffith University, AUSTRALIA

## Abstract

**Aim:**

Previous studies have shown that children of caregivers with low oral health literacy (OHL) had more untreated caries than children of caregivers with adequate OHL. However, there is a paucity of information on this relationship among children and youth with special health care needs (CYSHCN). Accordingly, this study aims to assess the association between the caregivers’ OHL and the oral health status of CYSHCN.

**Methods:**

This cross-sectional study was conducted in four schools dedicated for CYSHCN. A 48-item questionnaire gathered information about the demographic and socioeconomic factors, the child/adolescent’s medical condition, dental characteristics, caregiver self-efficacy and the child’s dental attitude. The Comprehensive Measure of Oral Health Knowledge (CMOHK) questionnaire was used to assess the caregivers’ OHL. The Löe & Silness gingival index (GI) and the Silness & Löe plaque index (PI) were used to assess gingival health and plaque levels, respectively. Directed acyclic graphs (DAGs) were utilized for the selection of the appropriate set of confounding variables for regression analysis. The mean score differences and 95% confidence intervals (CI) were estimated to quantify the associations of the various covariates with oral health outcome variables.

**Results:**

This study included 214 child/caregiver dyads. Most participants were physically disabled (56.1%) followed by children with hearing difficulty (9.8%) and congenital anomalies/syndromes (7.9%). The mean PI and GI of the children was 1.26±0.52 and 1.30±0.47, respectively. The median CMOHK score was 12 and the respondents were dichotomized based on the median value. Low caregiver oral health conceptual knowledge was significantly associated with higher PI scores (β [95% CI] = -0.26 [-0.41, -0.13]; p<0.001. Older participants (12-21-year-olds) had significantly higher plaque scores compared with younger participants (6-12-year-olds) (β [95% CI] = 0.33 [0.18, 0.51]; p<0.001). Participants who brushed their teeth twice or more daily had significantly lower (β [95% CI] = -0.15 [-0.43, -0.01]; p = 0.046). Conceptual knowledge score was not significantly associated with GI.

**Conclusion:**

This study found lower caregiver OHL levels to be associated with higher plaque scores for their child.

## Introduction

Oral health literacy (OHL) is “the degree to which individuals have the capacity to obtain, process, and understand basic oral health information and services needed to make appropriate health decisions” [1]. In the last two decades, OHL has increasingly gained the attention of oral health practitioners, researchers, and policy makers due to its proven impact on oral health outcomes [2]. The initial studies on OHL primarily focused on work recognition (Rapid Estimate of Adult Literacy in Dentistry -REALD [3]) or the reading comprehension (Test of Functional Health Literacy in Adults -TOFHLA [4]) skills of the respondents. However, these measures ignore the fact that individuals get information from a variety of sources, including written materials, audio-visual media, interpersonal interaction within the healthcare system, to name a few. The Institute of Medicine [5] in its landmark publication, Health Literacy: A Prescription to End Confusion, identified conceptual knowledge as one of the constructs of health literacy. Conceptual knowledge focuses assessing the general oral health knowledge, as well as specific knowledge of oral disease prevention and management. Macek *et al*. [6] presented a conceptual framework for the pathway between health literacy and oral health. According to this framework, oral health literacy encompasses four unique constructs: 1) word recognition, 2) reading comprehension, 3) conceptual knowledge, and 4) communication skills. The authors reported that conceptual knowledge provides a more suitable means of assessing how well an individual might understand, appraise and apply health information, as compared with assessing an individual’s reading comprehension or word recognition skills. The authors developed an instrument to measure the oral health conceptual knowledge, called the Comprehensive Measure of Oral Health Knowledge (CMOHK) [6]. Researchers have encouraged the use of conceptual knowledge instrument for studies pertaining to beliefs and self-efficacy in oral health [7].

In healthy individuals, poor OHL has been associated with poor oral health status, deleterious oral health behaviors, poor patient compliance and poor utilization of health care services [2, 8–19]. Recently, Zhou *et al*. [20] reported that parents with poor OHL had less compliance with oral health education and follow-up appointments for their preschool children with special health care needs (SHCN).

The American Academy of Pediatric Dentistry (AAPD) defines SHCN as “any physical, developmental, mental, sensory, behavioral, cognitive, or emotional impairment or limiting condition that requires medical management, health care intervention, and/or use of specialized services or programs” [21]. Among this population, oral diseases can have a direct and debilitating effect on the general health and quality of life of the individuals [21, 22]. Previous reports among children and youth with SHCN (CYSHCN) revealed that this population has significantly poorer oral hygiene, higher caries incidence and unmet restorative needs and severe periodontal disease compared with other children [13, 23–25]. CYSHCN might be at an increased risk for the development of oral diseases due to their compromised motor, sensory and intellectual abilities [26]. Maintaining good oral hygiene for CYSHCN is a challenge as they tend to have poor control of lips or tongue, inadequate motor capacity and poor cognitive abilities to understand the importance of oral health [27–29]. A recent study using data from the 2016–2018 National Survey of Children’s Health (NSCH) in the United States, reported that CYSHCN have poorer oral health compared with non-CYSHCN despite receiving more preventive oral health services [30]. CYSHCN rely more on their parents or caregivers to support their daily living activities [31]. Therefore, it may be assumed that the caregiver’s health literacy, attitude and expectations about oral health and oral hygiene practices would have a greater influence the oral health of CYSHCN than for others [32].

Though the body of dental literature linking OHL to oral health status continues to grow, far less is known about the influence of OHL on oral health status of CYSHCN. The present study is based on the null hypothesis that caregivers’ OHL is not associated with the oral health status of CYSHCN. Accordingly, this study aims to assess the association between the caregivers’ OHL and the oral health status of CYSHCN.

## Materials and methods

### Study setting and sampling procedure

This study was conducted between October 2019 and December 2020. Kuwait is divided into six different administrative areas (Governorates). A list of all the schools dedicated to CYSHCN across the six Governorates in Kuwait were compiled from the Public Authority for Disability Affairs (PADA). The sampling frame comprised of 40 schools dedicated to CYSHCN in Kuwait (14 schools in Hawalli Governorate, 10 schools in Farwaniya Governorate, five schools in Ahmadi Governorate, 4 schools each in Al-Asimah and Jahra Governorates and three schools in Mubarak Al-Kabir Governorate). Nursery schools catering to children less than 6 years were excluded. A two-stage cluster sampling technique was used for the selection of sample. For the first stage, four schools were randomly selected from sampling frame, representing 10% of all schools dedicated to CYSHCN in Kuwait. In the second stage, the selected schools were contacted for information on the class strength. Based on the reported class strength, it was decided to randomly select four to five classes from each school between grades 1 to 12 to match with the a priori sample size estimates. All the students in the selected classes were invited to participate in the study. The following selection criteria were employed for the inclusion of subjects: (1) CYSHCN as defined by AAPD [21]; (2) 6–21 years old; and (3) enrolled in the selected SHCN school. Students (1) who received professional dental prophylaxis within the previous three months; (2) undergoing fixed orthodontic treatment; (3) with severe debilitating medical condition; (4) who were taking medications are known to induce gingival changes; and (5) for whom an oral examination could not be performed, were excluded from this study. The questionnaires as well as the consent forms were sent to the caregivers of the selected students. For non-respondents, a second reminder was sent two weeks later.

### Power analysis

A power assessment for linear regression was based on using plaque/gingival scores as the continuous outcome variable and dichotomized health literacy scores as predictor variable. Sample size calculation was performed by G*Power 3.1.9.7 [33]. Assuming a moderate effect size of 0.13 [34] for a model with six predictor variables, it was estimated that with the inclusion of at least 112 subjects, the study will achieve 80% power with a 0.05 two-sided significance level. The final required sample size was fixed at 200 subjects to account for the design effect of cluster sampling. Assuming a conservative 50% response rate, it was decided to invite 100 students from each of the four selected schools to participate in the study.

### Data collection

The study team visited the schools and distributed the questionnaire with the informed consent forms to the students in the selected classrooms. Caregivers were contacted and the study objectives were explained. Those caregivers who agreed to participate in the study were asked to sign the informed consent form and complete the questionnaire. The 48-item questionnaire gathered information about the demographic and socioeconomic factors (age, gender, marital status, monthly income, educational level, area of residence and their relationship to the child), the child’s medical condition (diagnosis of the child’s condition, the caregiver perception of its severity and number of hospitalizations in the preceding year) and the child’s dental characteristics (last dental visit, treatment received in the previous visit and frequency of brushing).

Caregivers were asked to answer 5 self-efficacy items that were adopted from the Dental Self-Efficacy Scales by Syrjälä *et al*. [35] and Self-Efficacy Scale described by Kakudate *et al*. [36]. A 4-point Likert-type scale was used to record the responses, which ranged from ‘well prepared (+ +)’, ‘moderately prepared (+)’, ‘a little prepared (-)’ and ‘not at all prepared (- -)’. A dichotomous variable was created for each question by merging the affirmative responses (+ +/+) and non-affirmative responses (- -/-). Affirmative responses were scored of 1 and non-affirmative responses were scored 0. The five items of self-efficacy were summed up to get the total score (Range 0 to 5). The child’s dental attitude during examination was assessed using the Frankl Behavior Scale [37]. Dental attitude was dichotomized into positive (definitely positive and positive) and negative (definitely negative and negative) categories.

The main independent variable was the caregivers’ OHL. The Comprehensive Measure of Oral Health Knowledge (CMOHK) questionnaire was used to assess the caregivers’ OHL [6]. CMOHK questionnaire consists of 23 questions: 10 questions assessing the respondents’ basic knowledge, six questions assessing knowledge about dental caries prevention and management, five questions assessing knowledge about periodontal disease prevention and management, and two questions assessing oral cancer prevention and management. A previously validated Arabic version of CMOHK (CMOHK–A) questionnaire was used in this study [15]. The correct response for each CMOHK item was given a score of 1 and wrong answer was scored 0. We computed a cumulative score ranging from 0 (least conceptual knowledge level) to 23 (highest conceptual knowledge level) and estimated Cronbach-α as a measure of internal consistency and reliability. Subjects were then dichotomized into those with adequate (> the median score) and low (≤ the median score) conceptual knowledge levels [9, 15].

### Clinical examination

The children were examined at the dental clinics of the SOHP by two trained and calibrated dentists. Prior to the study, the examiners were calibrated for accuracy and repeatability using the Löe & Silness gingival index (GI) [38] and Silness & Löe plaque index (PI) [39]. GI and PI were assessed on all teeth present in the mouth and the total score were computed by adding up all the scores by tooth and then dividing them by the number of teeth. Total individual scores ranged from 0 (healthy) to 3 (disease). Training and calibration of the examiners were conducted by two experienced specialists (JKB and MAQ) at the Faculty of Dentistry, Kuwait University. The inter- and intra-examiner reliability was assessed using Kappa statistics and the values obtained was >80% for both GI and PI, indicating excellent reliability. A mouth mirror and a standardized periodontal probe (CP11; Hu-Friedy, Chicago, IL, USA) were used for the examination.

### Directed Acyclic Graphs (DAGs)

**Fig 1:**
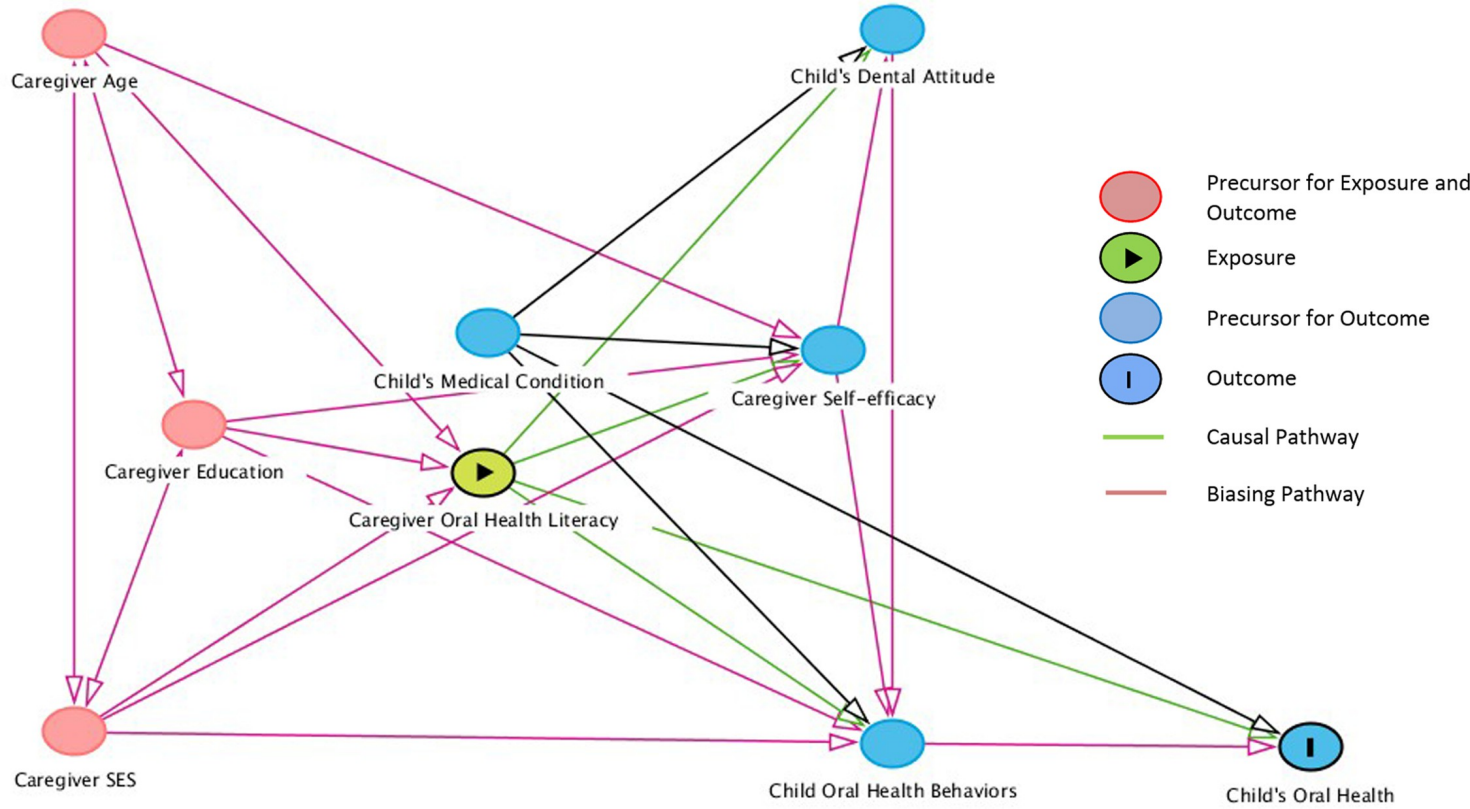
Directed acyclic graphs (DAG) showing the associations between exposure (oral health literacy) and outcome (oral health) specifying the relationships among the covariates.

The selection of appropriate set of confounding variables were made utilizing directed acyclic graphs (DAGs). Based on causal diagram theory, DAGs are considered sets of arrows that characterize causal associations between exposures and outcomes and also specify relationships among other variables that influence the exposure or outcome [40]. The nodes on the DAGs represent the variables and the arrows represent the paths. The front door paths have arrowheads pointing from exposure to the outcome representing the presence of causal effects. Backdoor paths are noncausal biasing paths between exposure and outcome. Backdoor paths may confound a direct effect between exposure and outcome when left open [41]. In Fig 1, the socio-demographics and the child’s medical condition were considered as a priori confounders for the association between caregivers’ OHL levels and the child’s oral health. The caregivers’ self-efficacy, the child’s dental attitude and the child’s oral health behaviors (dental utilization and frequency of brushing) were considered as effect mediators in the association between the caregiver’s OHL and their child’s oral health.

### Data management and analysis

Normality assumption for GI, PI and OHL was tested using the Kolmogorov-Smirnov (K-S) test and the Shapiro-Wilk test [42]. The multivariable regression model for the outcome variables GI and PI were built using the backwards procedure for variable selection with a p<0.1 criterion. Covariates were included in the model if they improved the maximum adjusted R square estimate of the effect of literacy on the outcome variables (GI and PI). The models were adjusted for selected socio-demographic variables, medical condition, oral health behaviors, dental attitude, self-efficacy and OHL. Pairwise correlations did not exceed 80% and both mean and individual variance inflation factors approximated 1, indicating no signs of serious multicollinearity. The DAGitty (http://www.dagitty.net/) online tool was used to check the postulated DAGs for consistency and validity of the minimum adjustment sets. Chi-square and Fisher’s exact tests was used to test the significance of associations in cross tables. Means between groups were assessed using Student’s t-test or ANOVA. The mean score differences between the reference group and each variable’s response level, the 95% confidence intervals (CI) and the covariates significance levels under the multivariable models are presented. The level of significance was set to p<0.05. All bivariate and multivariable analyses were carried out using SPSS 27.0 (IBM Corp. Released 2017. IBM SPSS Statistics for Windows, Version 27.0. Armonk, NY: IBM Corp.).

### Ethical aspects

The Ethics Committee of Kuwait University at the Health Science Center (HSC) approved this study (VDR/EC/3329; Dated: June 3, 2018). Permission to conduct the study in the selected schools was obtained from the Ministry of Health, the Ministry of Education and from the concerned authorities at the selected schools. This study is reported in accordance with the STrengthening the Reporting of OBservational studies in Epidemiology (STROBE) statement [43].

## Results

A total of 485 children, aged 6–21 years, were identified by the study team as potentially eligible for the study and consent forms along with the questionnaire were sent to their caregivers. Among these, 237 children returned the signed informed consent form and the completed questionnaires.

Case-wise deletion method was adopted for handling missing data in CMOHK. This resulted in deletion of 23 responses. Finally, data from 214 students were analysed.

Cronbach α for CMOHK was 0.83. The mean PI and GI of the children was 1.26±0.52 and 1.30±0.47, respectively. The median CMOHK score was 12 (interquartile range: 8–14) and the respondents were dichotomized based on the median value. Table 1 shows the results of the bivariate analysis of the distribution of the study sample in relation to the two oral health literacy groups: There were almost 40% of caregivers in the age group of 30-40-year-old age group and 40-50-year-old age group. A significantly (p = 0.033) higher percentage of female caregivers had adequate OHL levels (53.1%) as compared with male caregivers’ (37.3%). None of the other child/caregiver characteristics were significantly different between the two groups.

**Table 1:** Bivariate comparison for the demographics, medical conditions, dental characteristics between caregivers with low and adequate levels of oral health literacy.

Variables	All Subjects	Low OHL	Adequate OHL	
	N (%)	(< = 12) N (%)	(>12) N (%)	p-value*
**Total**	214 (100.0)	111 (51.9)	103 (48.1)	
**Caregiver Characteristics**				
**Age in years**	42.68±8.02	43.3±8.78	42.01±7.10	0.242
Less than 30	9 (4.2)	6 (66.7)	3 (33.3)	
31–40	87 (40.7)	42 (48.3)	45 (51.7)	0.351
41–50	85 (39.7)	42 (49.4)	43 (50.6)	
Greater than 51	33 (15.4)	21 (63.6)	12 (36.4)	
**Relationship with the child**				
Mother	147 (68.7)	69 (46.9)	78 (53.1)	0.033
Father	67 (31.3)	42 (62.7)	25 (37.3)	
**Marital Status**				
Married	188 (87.9)	94 (50.3)	94 (50.0)	
Divorced/Widow	26 (12.1)	17 (65.4)	9 (34.6)	0.15
**Monthly Household Income (USD)**				
Less than 1650	21 (9.8)	9 (42.9)	12 (57.1)	
1650 to 3300	44 (20.6)	22 (50.0)	22 (50.0)	0.684
3301 to 6600	89 (41.6)	50 (56.2)	39 (43.8)	
More than 6600	60 (28.0)	30 (50.0)	30 (50.0)	
**Educational Level**				
Less than high school	83 (38.8)	47 (56.6)	36 (43.4)	
High School	40 (18.7)	15 (37.6)	25 (62.5)	0.075
Bachelor	70 (32.7)	41 (58.6)	29 (41.4)	
Master and above	21 (9.8)	8 (38.1)	13 (61.9)	
**Participant’s Demographics**				
**Age in years**	12.90±3.40	13.17±3.6	12.6±3.16	0.22
6–12	99 (46.3)	49 (49.5)	50 (50.5)	0.306
13–21	115 (53.7)	62 (53.9)	53 (46.1)	
**Gender**				
Female	51 (23.8)	23 (45.1)	28 (54.9)	0.267
Male	163 (76.2)	88 (54.0)	75 (46.0)	
**Participant’s Medical Condition**				
ADHD/Intellectual Disability	9 (4.2)	5 (55.6)	4 (44.4)	
Learning Disability	3 (1.4)	2 (66.7)	1 (33.3)	
Muscular Dystrophy	14 (6.5)	4 (28.6)	10 (71.4)	
Asthma	15 (7.0)	6 (40.0)	9 (60.0)	
Autism / Cerebral Palsy	11 (5.1)	5 (45.5)	6 (54.6)	
Hearing Difficulty	21 (9.8)	10 (47.6)	11 (52.4)	
Physical Disability	120 (56.1)	71 (59.2)	49 (40.8)	
Congenital Anomalies/Downs Syndrome	17 (7.9)	7 (41.2)	10 (58.8)	
Metabolic Disorders	4 (1.9)	1 (25.0)	3 (75.0)	
**Caregivers perception of the severity of medical condition** ^**#**^				
Mild	35 (18.8)	14 (40.0)	21 (60.0)	0.402
Moderate	48 (25.8)	26 (54.2)	22 (45.8)	
Severe	103 (55.4)	53 (51.5)	50 (48.5)	
**Participant’s hospitalization history in the preceding year** ^**#**^				
None	162 (85.7)	81 (50.0)	81 (50.0)	0.679
One or more times	27 (14.3)	15 (55.6)	12 (44.4)	
**Participant’s Dental Characteristics**				
**Last Dental Visit**				
Within the last 6 months	103 (48.1)	53 (51.5)	50 (48.1)	0.494
Between 6 months– 1 year	63 (29.4)	36 (57.1)	27 (42.9)	
More than a year back	48 (22.4)	22 (45.8)	26 (54.2)	
**Reason for the previous dental visit**				
Routine check-up	80 (37.4)	43 (53.8)	37 (46.3)	0.422
Extraction	32 (15.0)	17 (53.1)	15 (46.9)	
Restoration	43 (20.1)	24 (55.8)	19 (44.2)	
Prophylaxis	47 (22.0)	24 (51.1)	23 (48.9)	
Root canal treatment	12 (5.6)	3 (25.0)	9 (75.0)	
**Frequency of toothbrushing**				
Less than twice daily	90 (42.1)	48 (53.3)	42 (46.7)	0.41
Twice daily or more	124 (57.9)	63 (50.8)	61 (49.2)	
**Participant’s Dental Attitude** ^#^				
Positive	126 (62.1)	68 (54.0)	58 (46.0)	0.318
Negative	77 (37.9)	36 (46.8)	41 (53.2)	

*Pearson Chi-square statistics.

^**#**^ Missing values present.

OHL–Oral health literacy.

Most of the caregivers were married (87.9%) and about 40% had a monthly household income within the range of 3,300 to 6,600 US dollars. A high percentage of caregivers had less than high school education (38.8%) and about a third of the respondents had a bachelor’s degree. The mean age (SD) of the children was 12.9 (3.40) years and about three-fourths were boys (76.2%). They were categorized into two age groups: 6-12-year-olds (46.3%) and 13-21-year-olds (53.7%). Most of the children were physically disabled (56.1%) followed by children with hearing difficulty (9.8%) and congenital anomalies/syndromes (7.9%). More than half of the caregivers perceived the child’s medical condition to be ‘severe’, and about 14% of the children had been hospitalized due to their medical condition in the preceding year. About half of the children had a dental visit within the previous six months and about 40% of the visits were for routine dental check-up. Most of the children brushed twice or more daily (57.9%) and had a positive dental attitude (62.1%).

Table 2 presents the self-efficacy of caregivers with low OHL and adequate OHL. A significantly higher percentage of caregivers with adequate OHL levels reported being confident of getting their child the necessary medical attention (p = 0.048) and obtaining the necessary information to care for their child (p = 0.001) as compared with those with low OHL. The caregivers with adequate OHL had a significantly (p = 0.019) higher mean self-efficacy score compared with those with low OHL (2.22±1.02 and 1.87±1.01, respectively).

**Table 2:** Association between caregivers’ oral health literacy and self-efficacy.

Variables	All Subjects	Low OHL	Adequate OHL	
	N (%)	(< = 12) N (%)	(>12) N (%)	p-value*
How confident are you that you can…				
1. …get your child the required medical care				
Very confident	90 (45.0)	40 (44.4)	50 (55.6)	**0.048**
Not confident	110 (55.0)	63 (57.3)	47 (42.7)	
2. …get your child the required dental care				
Very confident	165 (80.1)	83 (50.3)	82 (49.7)	0.313
Not confident	41 (19.9)	23 (56.1)	18 (43.9)	
3. …control the child’s daily sugar intake				
Very confident	57 (27.1)	27 (47.4)	30 (52.6)	0.316
Not confident	153 (72.9)	80 (52.3)	73 (47.7)	
4. …obtain the necessary health information to take care of your child				
Very confident	44 (20.8)	13 (29.5)	31 (70.5)	**0.001**
Not confident	168 (79.2)	96 (57.1)	72 (42.9)	
5. …choose the right health care provider to meet your child’s healthcare needs				
Very confident	47 (22.1)	24 (51.1)	23 (48.9)	0.530
Not confident	166 (77.9)	86 (51.8)	80 (48.2)	
**Self-efficacy (Mean**±**SD)**	2.05±1.03	1.87±1.01	2.22±1.02	**0.019**

*Pearson Chi-square statistics.

OHL–Oral health literacy.

The association between the various covariates and the outcome variable is represented as DAGs (Fig 1).

After adjusting for the other covariates in the multivariable linear regression model, the caregiver’s OHL was significantly associated with PI scores (β [95% CI] = -0.26 [-0.41, -0.13]; p<0.001) (Table 3). As compared with families with less than 1650 USD monthly income, children belonging to families with higher incomes had significantly lower PI scores (p<0.05). Older participants (12-21-year-olds) had significantly higher plaque scores compared with younger participants (6-12-year-olds) (β [95% CI] = 0.33 [0.18, 0.51]; p<0.001). Participants who brushed their teeth twice or more daily had significantly lower (β [95% CI] = -0.15 [-0.43, -0.01]; p = 0.046). The adjusted R square value for the model for PI was 20%. After adjusting the model for covariates, OHL was not significantly associated with GI (Table 4). As compared with younger participants, older participants had significantly higher GI scores (β [95% CI] = 0.30 [0.13, 0.43]; p<0.001). There was no significant association between the other covariates and GI. The variables in the model explained about 13% of the variance in the GI scores.

**Table 3:** Multivariable linear regression analysis for the association of plaque index with the various caregiver/child level factors.

Variables	Unadjusted		Adjusted		
	Mean±SD*	95% Confidence Interval	Standardized Coefficients Beta	95% Confidence Interval	p-value
**PLAQUE INDEX**					
**Oral health literacy**					
Low	1.40±0.48	Ref	Ref.	Ref.	
Adequate	1.10±0.52	**-0.43, -0.16** ^‡^	-0.26	**-0.41, -0.13** ^‡^	**<0.001** ^‡^
**Monthly Household Income (USD)**					
Less than 1650	1.32±0.52	Ref	Ref	Ref.	
1650 to 3300	1.25±0.50	-0.34, 0.20	-0.23	**-0.58, -0.03** ^**†**^	**0.03** ^**†**^
3301 to 6600	1.25±0.50	-0.32, 0.18	-0.28	**-0.55, -0.05** ^**†**^	**0.029** ^**†**^
More than 6600	1.26±0.56	-0.32, 0.20	-0.27	**-0.58, -0.06** ^**†**^	**0.015** ^**†**^
**Participant’s age in years**					
6–12	1.11±0.51	Ref	Ref	Ref	
12–21	1.39±0.49	**0.14, 0.41** ^‡^	0.33	**0.18, 0.51** ^‡^	**<0.001** ^‡^
**Frequency of daily brushing**					
Less than twice	1.31±0.47	Ref	Ref	Ref	
Twice or more	1.19±0.48	-0.33, 0.10	-0.15	**-0.43, -0.01** ^**†**^	**0.046** ^**†**^
**Age of Caregiver**					
Less than 30	1.32±0.48	-0.54, 0.22	0.02	-0.38, 0.45	0.862
30–40	1.13±0.53	**-0.56, -0.15** ^**†**^	-0.19	-0.43, 0.03	0.092^‡^
40–50	1.30±0.50	-0.40, 0.02	-0.11	-0.34, 0.10	0.291
Greater than 50	1.49±0.47	Ref	Ref	Ref	
**Self-efficacy Score**	1.30±0.46	-0.04, 0.11	0.08	-0.04, 0.12	0.308

Adjusted R^2^ for Plaque Index = 0.20.

*Independent sample t-test or ANOVA.

^**†**^ Statistically significant at the 5% level;

^‡^Statistically significant at the 1% level.

**Table 4:** Multivariable linear regression analysis for the association of gingival index with the various caregiver/child level factors.

Variables	Unadjusted		Adjusted		
	Mean±SD*	95% Confidence Interval	Standardized Coefficients Beta	95% Confidence Interval	p-value
**GINGIVAL INDEX**					
**Oral health literacy**					
Low	1.34±0.48	Ref	Ref	Ref	
Adequate	1.26±0.46	-0.21, 0.05	-0.08	-0.21, 0.06	0.283
**Participant’s age in years**					
6–12	1.15±0.50	Ref	Ref	Ref	
12–21	1.44±0.40	**0.16, 0.41** ^‡^	0.30	**0.13, 0.43** ^‡^	**<0.001** ^‡^
**History of Hospitalization in the last one year**					
None	1.31±0.47	Ref	Ref	Ref	
Yes	1.19±0.48	0.26, -0.31	-0.14	-0.38, 0.01	0.068^‡^
**Frequency of brushing**					
Less than twice daily	1.31±0.44	Ref	Ref	Ref	
Twice daily or more	1.30±0.49	-0.14, 0.11	-0.07	-0.20, 0.07	0.336
**Monthly Household Income (USD)**					
Less than 1650	1.32±0.36	Ref	Ref	Ref	
1650 to 3300	1.35±0.45	-0.22, 0.28	-0.09	-0.36, 0.07	0.420
3301 to 6600	1.26±0.48	-0.28, 0.17	-0.14	-0.37, 0.10	0.263
More than 6600	1.34±0.50	-0.21, 0.26	-0.09	-0.34, 0.15	0.449
**Age of Caregiver**					
Less than 30	1.25±0.54	-0.64, 0.04	-0.06	-0.55, 0.25	0.470
30–40	1.22±0.47	**-0.52, -0.15** ^**†**^	-0.17	-0.39, 0.06	0.141
40–50	1.30±0.48	**-0.44, -0.07** ^**†**^	-0.15	-0.36, 0.07	0.182
Greater than 50	1.55±0.35	Ref	Ref	Ref	

Adjusted R^2^ for Gingival Index = 0.13.

*Independent sample t-test or ANOVA.

^**†**^ Statistically significant at the 5% level; `

^‡^Statistically significant at the 1% level.

## Discussion

This study is the first to our knowledge to report on the relationship between caregivers’ oral health conceptual knowledge, sociodemographic characteristics, personality traits and the oral health status of CYSHCN. The results of this study suggest that caregiver characteristics like low oral health conceptual knowledge and low socioeconomic status; and, child level characteristics like older-age-group and lower frequency of brushing are strongly associated with higher plaque scores. In addition, older age group subjects (>12 years) had more gingival inflammation compared with younger children.

CMOHK was found to be a valid and reliable measure of OHL to detect statistical associations with clinical measures of periodontal health including plaque scores [44]. The Arabic translated version of CMOHK (CMOHK-A) has been previously validated in a similar population [15]. The CMOHK was found to be better suited for differentiating health literacy levels at the lower end of the scale than REALD or TOFHLA [6]. Previous research [7] have shown that conceptual knowledge was significantly related to dental beliefs, attitudes and self-efficacy, thereby confirming the suitability of using CMOHK as an OHL measure in this study. In the original study, Macek *et al*. [6] categorized the respondents CMOHK scores into three categories: scores from 0 to 11 represented ‘poor’, scores from 12–14 represented ‘fair’, and scores from 15–23 represented ‘good’ OHL levels. However, to date there are no norms established to indicate a score for ‘adequate’ OHL. As in a previous investigation [15], the median score was used to categorize the respondents into ‘low’ and ‘adequate’ conceptual knowledge level groups. In this study, children of caregivers with higher levels of oral health conceptual knowledge had lower plaque scores. Oral health conceptual knowledge directly results from interacting with dentists/dental hygienists and individuals with higher levels of oral health conceptual knowledge might be more likely to visit a dentist/dental hygienist because they might be more aware of the importance of oral health.

Previous research has shown that CMOHK is associated with the respondents confidence in filling out forms [7]. Since CMOHK was able to assess the functional skill set of the caregivers, it provides additional evidence of its applicability as a valid measure of OHL [7]. The health seeking behavior is influenced by a number of factors, including the individual’s perception about the symptoms, perceived disrespect, and ability to understand the health care system [45]. Since OHL plays a central role in one’s ability to recognize oral health information and act upon it, it may affect the caregivers’ perception of their children’s oral status, as well as their healthcare seeking behavior.

Studies comparing individuals with disabilities to similarly aged individuals with normal development have shown poorer oral hygiene and increased periodontal disease within the disability group [46]. CYSHCN may have poorer oral health due to their underlying medical condition which puts them at higher risk and/or due to caregiver level factors like not being able to recognize a need. CYSHCN have chronic dependence on caregivers for their oral as well as general health. The caregiver’s knowledge and attitude towards the child’s oral health plays a vital role in ensuring that the child receives timely and routine dental care.

This study reports lower caregiver conceptual knowledge to be consistently and independently associated with higher plaque scores for their child. Similar findings were reported in the study by Vann *et al*. [12], though their study was conducted in a low-income community using a different OHL instrument (REALD). Bridges *et al*. [13] also reported caregiver oral health literacy to be associated with their child’s oral health even after adjusting for the effect of various potential confounders. Baskaradoss *et al*. [15] found that children of caregivers with poor OHL levels had more untreated caries than children of caregivers with adequate OHL. These findings suggest that oral health literacy is a fundamental dimension that confers oral health impacts above and beyond education and socio-demographic characteristics.

Almost half of the children in this study had a dental visit within the previous six-months and in more than half of the visits, only routine dental check-up or dental prophylaxis was performed. This is an indication of the raised awareness about the importance of preventive dental visits. Pediatric and public health programs have always promoted preventive dental care as the “cornerstone of optimal oral health promotion” [47]. Preventive dental care is also cost-effective and routine preventive and restorative services can reduce the need for more expensive emergency or inpatient treatment of dental problems.

Recognizing the benefits of focusing on of early prevention of dental diseases, the Ministry of Health, Kuwait, have implemented one of the largest SOHP in the world. SOHP is a comprehensive school-based oral health program with educational, preventive and treatment components serving the oral health needs of more than 300,000 school children between the age group of 6–16 years [22]. Schools for CYSHCN have been included to this program and most of these schools have dental clinics within their campuses. These initiatives help in raising the awareness among the caregivers on the importance of good oral health.

Lee *et al*. [48] found self-efficacy to act as a mediator or modifier for the relationship between OHL and oral health status. Studies by Jonsson *et al*. [49] showed that approaches targeting factors regarding the self-efficacy can improve oral hygiene behavior. This relationship was explored in this study. The general self-efficacy questionnaire [50] is popularly used in dental researches. However, previous research has shown that this instrument does not show significant correlation with oral hygiene indices [51]. It has been suggested that it may not be possible to predict better oral hygiene from a high general self-efficacy. Instead, it is important to measure task specific self-efficacy [52]. Therefore, task specific self-efficacy questions matching our study objectives were developed for this research. In this study, caregivers with low conceptual knowledge scores had significantly lower self-efficacy scores compared with those with adequate conceptual knowledge scores. Though the association between self-efficacy scores and plaque scores were not statistically significant, it was added to the final models as it improved the predictive value of the final model.

In this study older age group subjects had significantly more plaque and gingival inflammation compared to younger age group subjects. It was indistinguishable whether the difference in oral health between younger and older age group subjects were due to the child level (e.g., child’s chronic illness or oral health behaviors) or more related to the caregiver level factors. It could be that the caregivers give more attention to the oral hygiene practices of younger children as compared to older children. This could also be due to the higher focus given by the SOHP on the oral health of younger children with regards to placement of dental sealants in this group. Our analysis did not differentiate preventive services from other dental treatments and therefore was not included in the final model.

The results of this study suggest children who brushed twice or more daily have lower levels of plaque. However, the frequency of brushing was not associated with the caregiver’s conceptual knowledge levels. This is in contrast to the results of Vann *et al*. [12], which reported lower caregiver literacy to be strongly associated with no daily tooth brushing. There are several factors that may explain the difference in the study findings between the two studies. Vann *et al*. [12] included only infants (less than 5-years-old) in their study, whereas, the current did not include this age group. The dependence on the caregiver for oral hygiene is much more higher for infants than for any other age groups. Additionally, the difference in the data collection method employed by the two studies could also have contributed to the contrasting results. Data collection for the study by Vann *et al*. [12] was performed by trained examiners using an interview schedule as compared with the current study, which used self-administered questionnaires.

The present results should be considered in light of the study’s limitations. Firstly, the cross-sectional design of the study limits the potential to make any causal inferences with regard to the pathways that may link caregiver conceptual knowledge levels with the child’s oral health. Secondly, this study used a non-probability sampling technique for selection of study participants, thus limiting the generalizability of the results. Thirdly, as with all the investigations examining health literacy, it is acknowledged that low caregiver conceptual knowledge levels may be a threat to the study’s validity because of the caregiver’s difficulty in reading and comprehending survey questions. The caregiver’s with low levels of conceptual knowledge are likely to have difficulty completing the questionnaire or giving informed consent to participate in this study. It was not feasible to assess if the caregiver characteristics differed between those who agreed to participate in the study and those who did not. Finally, influence of culture might have shaped the caregivers’ perspectives, negotiating these opinions culturally was not within the scope of this study.

To conclude, this study found lower caregiver OHL levels to be associated with higher plaque scores for their child. The results of this study could help in planning awareness campaigns to improve the oral health knowledge and attitude of the caregiver. Integrating literacy-based approaches to existing initiatives for caregivers may help to improve the oral hygiene of CYSHCN.

## Supporting information

S1 Data (SAV)
