# Correction: Association between the caregivers’ oral health literacy and the oral health of children and youth with special health care needs

**DOI:** 10.1371/journal.pone.0314904

**Published:** 2024-11-27

**Authors:** Jagan Kumar Baskaradoss, Aishah AlSumait, Eman Behbehani, Muawia A. Qudeimat

There are errors in the Author Contributions. The correct contributions are:

**Conceptualizatio**n: Jagan Kumar Baskaradoss, Muawia A. Qudeimat, Eman Behbehani

**Data curation:** Jagan Kumar Baskaradoss

**Formal analysis:** Jagan Kumar Baskaradoss

**Funding acquisition:** Jagan Kumar Baskaradoss, Muawia A. Qudeimat, Eman Behbehani

**Investigation:** Jagan Kumar Baskaradoss, Aishah AlSumait

**Methodology:** Jagan Kumar Baskaradoss, Aishah AlSumait, Eman Behbehani, Muawia A. Qudeimat

**Project administration:** Jagan Kumar Baskaradoss

**Resources:** Jagan Kumar Baskaradoss

**Validation:** Jagan Kumar Baskaradoss

**Writing–original draft:** Jagan Kumar Baskaradoss

**Writing–review & editing:** Jagan Kumar Baskaradoss, Muawia A. Qudeimat, Eman Behbehani

## References

[1] Baskaradoss JK, AlSumait A, Behbehani E, Qudeimat MA (2022) Association between the caregivers’ oral health literacy and the oral health of children and youth with special health care needs. PLoS ONE 17(1): e0263153. 10.1371/journal.pone.0263153 35085332 PMC8794213

